# Is Perceived Exposure to Mosquitoes Associated with Actual Exposure? Results from Studies in High-Risk and Low-Risk Geographic Areas

**DOI:** 10.4269/ajtmh.19-0074

**Published:** 2019-09-23

**Authors:** Béatrice Gaillard, Fréderic Simard, Laurent Dormont, Pierre Jay-Robert, Denis D’Abadie de Lurbe, Manuel Etienne, Anne Baudin, Jocelyn Raude

**Affiliations:** 1 CEFE/Univ Paul Valéry Montpellier 3/CNRS/Univ Montpellier/EPHE/IRD/Montpellier, France;; 2 MIVEGEC/IRD/CNRS/Montpellier University, Montpellier, France;; 3 CEDRE Martinique;; 4 EID Mediterranée;; 5 UVE: Aix-Marseille Univ-IRD 190-Inserm 1207-IHU Méditerrannée Infection, Marseille, France

## Abstract

Perceived exposure to mosquitoes plays a fundamental role in the adoption of a range of protective behaviors aiming to prevent and control mosquito-borne disease. However, it is largely unknown in the present literature to what extent perceived exposure is associated with actual exposure. Moreover, the perception of nuisance may depend on the natural environment in which human populations are living, and especially its epidemiological context. In this study, the hypothesis that perceived exposure is driven by mosquito abundance was tested in two different geographic areas. We compared a range of perceived nuisance measures—collected through questionnaires—with egg number measured within ovitraps located in the south of France, which has been recently colonized by an arbovirus vector, and La Martinique island, a tropical French territory, which has a long history of outbreaks of mosquito-borne pathogens. Unexpectedly, only the nuisance due to mosquito noise was correlated with ovitrap activity in southern France. All other perceived exposure measures, both in the south of France and in Martinique, were not correlated with egg number surrounding households investigated. These results suggest the existence of habituation effects that may disturb the engagement in adaptive behaviors in the face of change in the entomological conditions.

## INTRODUCTION

Mosquito-borne pathogens are a growing concern worldwide.^1^ However, the development of insecticide resistance has dramatically decreased the effectiveness of conventional methods. Therefore, alternative methods of vector control, in particular those that promote behavior change in the public, are increasingly recognized as crucial in the prevention and control of these pathogens.^2^ However, as health protective behaviors are known to be very sensitive to the perceived exposure to vector, it is extremely important to know whether it adequately reflects the actual exposure of human population to mosquitoes.^3^

From an entomological point of view, abundance of adult mosquitoes (through BG traps or CDC traps mainly), egg abundance (through ovitraps), and composite indicators measuring area productivity (such as Breteau index) are commonly used. However, they are challenging to implement routinely in the field at large scales and require regular visits by trained staff.^4^ To evaluate the perceived exposure to mosquitoes, a common option is to conduct interviews among household inhabitants to measure the perceived nuisance generated by mosquitoes. Nuisance is a feeling generally characterized by an embarrassment or a suffering experience, which can be expressed by interviewed people according to the perception of this feeling. However, this perception can vary individually because this acoustic and kinesthetic perception tends to be highly subjective.^5^ Moreover, this nuisance feeling can also be modulated by the local epidemiological context, and especially the habituation effect of human population to mosquito-borne pathogen outbreaks.^6–8^

In this study, we aimed to understand in which context different measures of mosquito nuisance can be used as a reliable indicator of mosquito density. To test this assumption, we compare egg number and phone interviews, measuring different components of mosquito nuisance within different areas in the south of France (treated versus non-treated by social mobilization strategies), where mosquito presence is recent, and La Martinique island, where many mosquito-borne pathogens are endemic. It was also assumed that the epidemiological context may mitigate the association between the perceived and actual exposure to mosquitoes, with people in southern France being more sensitive to temporal variation in the actual exposure than people in La Martinique.

## METHODS

### Context.

Two localities in Martinique (Fort de France 14°36′57.833″N, 61°3′31.609″W, and Le Lorrain 14°49′36.793″N, 61°3′15.469″W) and in the south of France (Jacou 43°39′39.146″N, 3°54′41.231″E, and La Grande-Motte 43°33′38.534″N, 4°5′9.859″E) were selected because of their continuous entomological monitoring since years and received identical protocol to measure the perceived mosquito exposure. It is worth pointing out that these localities are homogeneous in socioeconomic terms (average income tax per household is 2,745 euros in Fort de France and 2,994 euros in Le Lorrain, and 3,314 euros in Jacou and 4,521 euros in La Grande-Motte). Within these territories, 188 households in Martinique (94 in Fort de France and 94 in Le Lorrain) and 350 households in the south of France (175 in La Grande-Motte and 175 in Jacou) have been randomly selected in such a way to ensure a distance of 50 m between each household. At the beginning of the study (in May 2017 in the south of France and in January 2018 in La Martinique), each household was visited by a mosquito control agent to obtain the informed consent to be involved in the study and for providing basic knowledge about mosquito life cycle and how to be protected.^9^ These two territories have received social mobilization treatment, that is, information about measures to decrease mosquito abundance and to avoid biting, ensuring therefore an heterogeneity in terms of mosquito abundance at the household scale.^10^ It is important to highlight that protection against mosquitoes mostly relies on our social mobilization treatment in the south of France, whereas households in Martinique have received more frequently visits from vector control services to decrease the number of breeding sites.

### Phone call contents.

At the end of the most favorable season for mosquitoes (December in the south of France and June in La Martinique), each household that has been involved in the study received a phone call to measure the intensity of different components of the nuisance perceived. These feelings of nuisance were measured through a set of five questions using an 11-point Likert response scale: “How do you evaluate the inconvenience caused by 1) mosquitoes outside the house, 2) mosquito bites, 3) mosquito noise at night, 4) the obligation to remove stagnant waters, and 5) the presence of mosquitoes in general?”

### Entomological surveys.

In parallel to the nuisance measured through phone interviews, ovitraps (measuring the number of mosquito eggs) have been uniformly placed within each locality to cover a reasonable number of households. In total, 40 ovitraps have been used in the two localities situated in the south of France (Jacou and la Grande-Motte) and 78 in the two localities of La Martinique (Le Lorrain and Fort de France). On average, each ovitrap covers 10 different households. These traps were collected every 2 weeks between May and September 2017 in the south of France and between January and April 2018 in La Martinique island.

### Statistical analyses.

First, Cronbach’s alpha coefficient was calculated to measure the consistency and reliability of the questions asked during the phone calls.^11^ This coefficient is designed to measure the internal coherence and the reliability of the questions asked during a test through quantifying the correlation between answers to questions related to the same topic (Cronbach’s coefficient is considered “acceptable” between 0.7 and 0.9).^11^ Then, simple Pearson correlation coefficients were calculated between the number of eggs recorded at every session and the perception of exposure based on the five questions at the end of the season, as well as with a composite index summarizing the answers to these questions (through adding the score of each question). We consider that our analysis scale is the household unit, and the corresponding number of eggs is the one quantified within the closest ovitrap. We applied a Bonferroni correction to consider multiple comparisons. All analyses were performed with the R software.^12^

## RESULTS

First of all, Cronbach alpha’s coefficient has been quantified at 0.83 (with a sample size of 137 over five items) in La Martinique and 0.78 (with a sample size of 137 over five items) in the south of France. Therefore, the answers produced by the different people interviewed are coherent and can be analyzed simultaneously. In La Martinique island (Table 1), the measure of perceived exposure was not found to be correlated with the number of eggs captured in surrounding ovitraps. In southern France (Table 2), only the self-reported noise produced by mosquitoes during the night in the south of France was partially correlated with the number of eggs during the months of July and August. No significant correlation with the composite index has been identified. Therefore, there is very poor association between entomological activity measured in ovitraps and nuisance indicators.

**Table 1:** *P*-values of Pearson correlation between nuisance measure and number of eggs measured within the nearest ovitraps in Martinique

	Nuisance due to mosquitoes outside	Nuisance due to mosquito bites	Nuisance due to night noise	Nuisance due to the requirement to remove stagnant water	Nuisance due to mosquito presence	Composite index
January 1	0.367	0.401	0.978	0.727	0.562	0.543
January 20	0.945	0.592	0.520	0.380	0.818	0.784
February 1	0.994	0.958	0.863	0.671	0.435	0.612
February 20	0.784	0.867	0.906	0.577	0.462	0.853
March 1	0.0942	0.259	0.536	0.742	0.122	0.676
March 20	0.734	0.732	0.163	0.340	0.767	0.312
April 1	0.630	0.837	0.799	0.512	0.610	0.578
Total	0.803	0.706	0.728	0.877	0.901	0.891

**Table 2:** *P*-values of Pearson correlation between nuisance measure and number of eggs measured within the nearest ovitraps in the south of France

	Nuisance due to mosquitoes outside	Nuisance due to mosquito bites	Nuisance due to night noise	Nuisance due to the requirement to remove stagnant water	Nuisance due to mosquito presence	Composite index
May 12	0.902	0.335	0.534	0.771	0.717	0.682
May 24	0.011	0.789	0.490	0.912	0.041	0.034
June 8	0.812	0.707	0.126	0.693	0.567	0.323
June 22	0.308	0.909	0.0497	0.469	0.904	0.481
July 5	0.340	0.576	**4.08e-4**	0.489	0.626	0.617
July 21	0.240	0.150	**1.89e-5**	0.247	0.089	0.092
August 3	0.767	0.454	0.001	0.289	0.109	0.641
August 17	0.007	0.034	0.335	1.75e-3	0.062	0.020
August 31	0.132	7.37e-4	**2.30e-4**	0.020	0.049	0.039
September 14	0.097	0.839	0.425	0.706	0.205	0.573
September 28	0.017	0.389	0.790	0.838	0.035	0.836
October 12	0.036	0.676	0.001	1.73e-3	0.680	0.531
Total	0.238	0.315	**7.81e-6**	0.254	0.778	0.214

Bold values represent significant correlations with a threshold at 6.41e-4 (after Bonferroni correction).

## DISCUSSION

In this study, we aimed to test whether the perceived nuisance measured by telephone surveys adequately reflects more objective indicators based on entomological surveillance. We found that only nocturnal noise is sometimes correlated with the mosquito egg number in the south of France, where mosquito presence is recent, but no components of nuisance is associated with egg numbers recorded in La Martinique, where outbreaks of mosquito-borne pathogens can be frequent. Therefore, our study provides strong evidence that nuisance indicators do not accurately reflect the entomological activity and, consequently, the epidemiological risk.

This lack of association between entomological activity and nuisance indicators shows a mismatch between the real activity and the one perceived by local population. An interesting possibility is that social pressure about the perception of mosquito presence at a regional or city scale, through informal discussion with personal relationships or local media coverage, could tend to homogenize the feeling about this health threat, independent of its local reality at a household scale. Nevertheless, such possibility can occur only when mosquito abundance has reached a sufficient threshold, to create such social pressure.

Regarding the small divergence between tropical and Mediterranean areas, a number of factors could explain this result. First, the ecological contexts are different.^13^ Indeed, in La Martinique, seasonality in mosquito abundance is pretty low, which can generate a continuous presence of mosquitoes that is only exacerbated during the rainy season. However, in metropolitan France, seasonality of mosquito population dynamics is much stronger with a complete disappearance during winter. Such difference could exacerbate the feeling of nuisance because the mosquito population is more concentrated through time. The second important difference is that the mosquito species involved are not the same. *Aedes albopictus*, which is implemented in the south of France, is known to be much more aggressive than *Aedes aegypti*, which is endemic in La Martinique.^14^ Consequently, the perceived nuisance could also be different. The last possibility is a different epidemiological history in these two territories. We are confronted with a logic of territory: a possible habituation effect in Martinique’s landscape versus a novelty that can be somewhat considered as a major threat to public health. In the south of France, the population has never experienced such an important bite pressure and has never been confronted with such an invasion of arthropods.^15^ In La Martinique, people have always lived with mosquitoes, producing a mosquito culture and psychosocial custom.^14^

Finally, the only nuisance indicator (mosquito noise during night) associated with entomological activity suggests not only mosquito presence within households during the night but also some kind of protection against mosquitoes (repellents or defensive behavior, for instance). Indeed, other nuisance measures, such as bite feeling, are not significantly associated with egg numbers. Therefore, people should not be frequently bitten because *Ae. albopictus* is known for its aggressive behavior and painful bites.^16^

This study has a certain number of limitations, in particular, because ovitraps, which are certainly attractive, are not necessarily a good indicator of adult mosquito presence. Indeed, these traps can compete with natural breeding sites, introducing a bias in the normal mosquito activity.^17^ Other measures could have been investigated, especially by considering the density of breeding sites in the houses visited, but it would have required a greater logistical effort at each sampling session. Therefore, we have privileged to consider a larger number of households and localities.

To conclude, although the results of our study can probably only apply within our specific contexts, it nevertheless highlights that using nuisance feelings as an entomological survey appears to have a limited interest. If this study has to be repeated in different contexts and with different entomological measures, the difference observed between our two territories also calls for considering carefully the specificity of the local context. Therefore, although we knew nothing about this match between perceived and actual mosquito exposure, our study clearly calls for keeping classical entomological monitoring even if this means to consider a smaller amount of localities and/or sampling sessions.
